# Generalized information criteria for personalized gene network inference

**DOI:** 10.3389/fgene.2025.1583756

**Published:** 2025-06-20

**Authors:** Heewon Park, Seiya Imoto, Sadanori Konishi

**Affiliations:** ^1^ School of Mathematics, Statistics and Data Science, Sungshin Women’s University, Seoul, Republic of Korea; ^2^ Data Science Center, Sungshin Women’s University, Seoul, Republic of Korea; ^3^ Human Genome Center, Institute of Medical Science, University of Tokyo, Bunkyo, Japan; ^4^ M&D Data Science Center, Institute of Science Tokyo, Tokyo, Japan; ^5^ Department of Mathematics, Faculty of Science and Engineering, Chuo University, Hachioji, Japan

**Keywords:** model evaluation, personalized gene network, generalized information criteria, acute myeloid leukemia, gastri cancer

## Abstract

Identifying individual genomic characteristics is a critical focus in personalized therapies. To reveal targets in such therapies, we considered personalized gene network analysis using kernel-based $L_{1}$ -type regularization methods. In kernel-based $L_{1}$ -type regularized modeling, selecting optimal regularization parameters is crucial because edge selection and weight estimation depend heavily on such parameters. Furthermore, selecting a kernel bandwidth that controls sample weighting is vital for personalized modeling. Although cross-validation and information criteria (i.e., AIC and BIC) are often used for parameter selection, such traditional techniques are computationally expensive or unsuitable for approaches based on estimation techniques other than maximum likelihood estimation. To overcome these issues, we introduced a novel evaluation criterion in line with the generalized information criterion (GIC), which relaxes the assumption of maximum likelihood estimation, making it suitable for personalized gene network analysis based on various estimation techniques. Monte Carlo simulations demonstrated that the proposed GIC outperforms existing evaluation criteria in terms of edge selection and weight estimation. Acute myeloid leukemia (AML) drug sensitivity-specific gene network analysis revealed critical molecular interactions to uncover ALM drugs resistant mechanism. Notably, PIK3CD activation and RARA/RELA suppression are crucial markers for improving AML chemotherapy efficacy. We also applied our strategy for gastric cancer drug sensitivity analysis and uncovered personalized therapeutic targets. We expect that the proposed sample specific GIC will be a useful tool for evaluating personalized modeling, including in sample characteristic-specific gene networks analysis.

## 1 Introduction

In recent years, significant attention has been paid to the identification of individual genomic characteristics, particularly with the growing focus on personalized therapy across various research areas, such as statistics, bioinformatics, and medical science. Heterogeneous genetic network analysis is attracting growing interest, as it provides crucial targets for personalized therapy because diseases are typically caused by perturbations in complex molecular interactions rather than by isolated genetic defects (Ahmed et al., 2020). Various computational and statistical methods have been developed to reveal the molecular interactions associated with disease mechanisms, such as Bayesian networks (Imoto et al., 2002), graphical lasso (Huang et al., 2020) and $L_{1}$ -type regularization (Zou and Hastie, 2005), among others. Although many strategies for gene network estimation have been developed and successfully applied in various fields of research, these strategies provide averaged gene network estimation results for all samples. That is, the existing methods cannot uncover sample (e.g., cell line and patient) characteristic-specific molecular interactions. Thus, we cannot effectively provide evidence for personalized therapy using these methods.

To address this issue, Shimamura et al. (2011) proposed the use of a kernel-based $L_{1}$ -type regularization method with a varying coefficient model (Hastie and Tibshirani, 1993), called NetworkProfiler. Park et al. (2019) developed a robust version of NetworkProfiler based on k-nearest neighbor-based bandwidth.

Kernel-based $L_{1}$ -type regularization strategies reveal molecular interactions under varying sample characteristics (e.g., drug sensitivity, cancer progression, survival time), enabling personalized gene network analysis. In kernel-based $L_{1}$ -type regularization for personalized gene network analysis, the selection of regularization parameters is essential because it plays a major role in determining edge selection and estimating edge weights. Additionally, selecting the bandwidth in the kernel function is crucial for sample-specific analysis because it determines the weights assigned to the samples in personalized modeling. However, relatively little attention has been paid to the evaluation of personalized modeling. Previous studies have selected the parameters and bandwidth using cross-validation (CV) or traditional information criteria, such as the Akaike information criterion (AIC) (Akaike, 1973) and Bayesian information criterion (BIC) (Schwarz, 1978). CV is computationally intensive, particularly in personalized gene network analysis, where n model estimations are required for n samples, leading to significant computational complexity. Furthermore, traditional information criteria are not applicable to kernel-based $L_{1}$ -type regularized regression modeling, because they were developed under the assumption that the model is estimated using the maximum likelihood method (Konishi and Kitagawa, 1996). To resolve these issues, we proposed a novel model evaluation criterion for personalized gene network analysis. We considered the generalized information criterion (GIC), which was derived by relaxing an assumption imposed on AIC; that is, that *The model is estimated by the maximum likelihood method*, and extended the GIC for sample-specific analysis. In the derivative of GIC, computation of the influence function is a crucial issue, where a second-order differentiable functional estimator is required. However, the functional estimator of the kernel-based $L_{1}$ -type regularization method cannot be derived analytically owing to indifferentiability of the $L_{1}$ -norm penalty. To address this problem, we referred to the local quadratic approximation of the $L_{1}$ -type penalty (Fan and Li, 2001). We then focused on the fact that the objective function of the kernel-based $L_{1}$ -type regularization method can be reformulated without a kernel function to derive a GIC for personalized gene network analysis. The proposed strategy enables us to evaluate a personalized model estimated using not only the maximum likelihood method, but also various other estimation methodologies.

**FIGURE 1:**
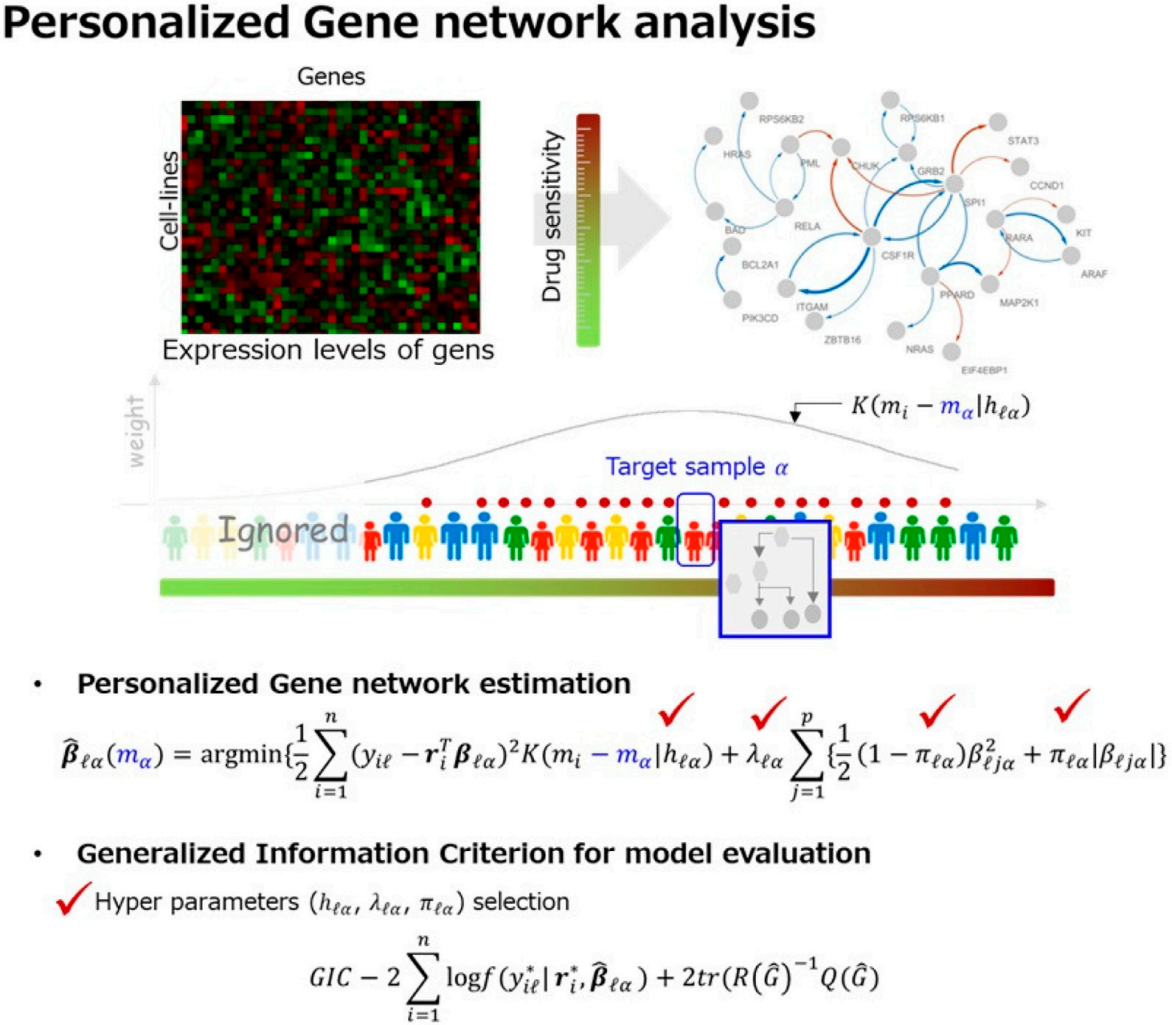
Overview of our strategy for personalized gene network analysis. By using the $L_{1}$ -type regularization method, we estimate personalized gene network based on characteristic of sample (e.g., drug sensitivity) and expression levels of genes. We then evaluate the estimated gene network (i.e., hyper parameters selection) by using the proposed sample-specific generalized information criterion (GIC).

Figure 1 shows schematic of the proposed strategy for personalized gene network analysis.

Monte Carlo simulations are conducted to illustrate the performance of the proposed strategy. The simulation results showed that the proposed GIC outperformed other model evaluation criteria for edge selection in a personalized gene network analysis. Furthermore, our strategy showed effective results for edge weight estimation. We applied the proposed GIC to the Sanger Genomics of Drug Sensitivity in Cancer (GDSC) dataset and performed drug sensitivity-specific gene network analysis for the FDA-approved acute myeloid leukemia (AML) drugs, i.e., doxorubicin, midostaurin, quizartinib, and cytarabine, where drug sensitivity is considered a characteristic of cell lines. In the AML drug sensitivity-specific gene network analysis, our strategy also showed effective results for network estimation. We then identified AML drug resistant- and sensitive-specific molecular interactions. Our results revealed the activity of PIK3CD and RARA/RELA in AML drug-sensitive- and resistant-specific molecular interactions. The identified markers were validated through literature as therapeutic targets for AML. Based on our findings and the existing literature, we suggest that suppression of the identified AML drug resistant-specific markers (i.e., RARA and RELA) and activation of the sensitivity-specific marker (i.e., PIK3CD) may offer essential guidance for improving chemotherapy.

The proposed strategy was also applied to dataset obtained from the Cancer Dependency Map (DepMap) Portal (https://depmap.org/portal/) and we performed gastric cancer drug sensitivity-specific gene network analysis. Our result uncovered FGF16, FGF6, CSNK1A1L and WNT1 as personalized therapeutic targets of gastric cancer.

Personalized medicine enables more precise treatments, early prevention strategies, patient-centered care, and potential cost reductions, which has driven extensive research efforts aimed at improving therapeutic outcomes across diverse medical fields. In statistics and computational biology areas, numerous studies have been conducted to provide data-driven evidences for personalized medicine. The kernel-based $L_{1}$ -type regularized regression modeling is one of approaches and have widely used to sample-specific analysis. In the sample-specific analysis based on the kernel-based $L_{1}$ -type regularized regression modeling, model evaluation (i.e., hyper parameters selection) is a crucial issue, because the model estimation and crucial features selection heavily rely on the hyper parameters values. However, there is a striking lack of research on evaluation of sample-specific model, even though model evaluation is also crucial for better understanding, interpreting model behaviors and further improving model performance. To the best of our knowledge, this is the first study on model evaluation criterion for sample-specific analysis. It was demonstrated that our strategy provides effective results for sample-specific analysis. We expect that the proposed sample-specific GIC will be a crucial tool of sample-specific analysis for personalized medicine. The remainder of this paper is organized as follows. In Section 2, we introduce a statistical model and estimation method for personalized gene network analysis. We introduce the proposed generalized information criterion in Section 3. The results of the Monte Carlo simulations are presented in Section 4. Finally, we describe the results of AML and gastric cancer drug sensitivity-specific gene network analysis in Section 5. The conclusions are presented in the Discussion section.

## 2 Methods

### 2.1 Personalized gene network analysis

Let $\left\{\left(y_{i\ell },r_{i}\right);i=1,\ldots ,n\right\}$ be a sample of i.i.d. random variables with a common distribution $G\left(y_{\ell },r\right)$ and density $g\left(y_{\ell },r\right)$ . We consider $r_{i}={\left(r_{i1},\ldots ,r_{ip}\right)}^{T}$ to be the expression levels of p regulator genes and $y_{\ell }={\left(y_{1\ell },\ldots ,y_{n\ell }\right)}^{T}$ to be the expression level of the $\ell^{th}$ target gene.

The following linear regression model is used to describe the molecular interactions between genes:

$$y_{i\ell }=r_{i}^{T}\beta_{\ell }+\epsilon_{i\ell },\;i=1,\ldots ,n,\;\ell =1,\ldots ,q,$$ (1)

where $\beta_{\ell }={\left(\beta_{\ell 1},\ldots ,\beta_{\ell p}\right)}^{T}$ is the regression coefficient vector that indicates the strength of the effect of p regulator genes on the $\ell^{th}$ target gene, and $\epsilon_{i\ell }∼N\left(0,\sigma^{2}\right)$ is the random error for the model of the $\ell^{th}$ target gene. Although the linear regression model in Equation 1 has been used to represent gene networks, it cannot describe sample (patient)-specific molecular interactions because it represents an averaged regulatory effect of p gene; that is, $\beta_{\ell }$ for all n samples.

**FIGURE 2:**
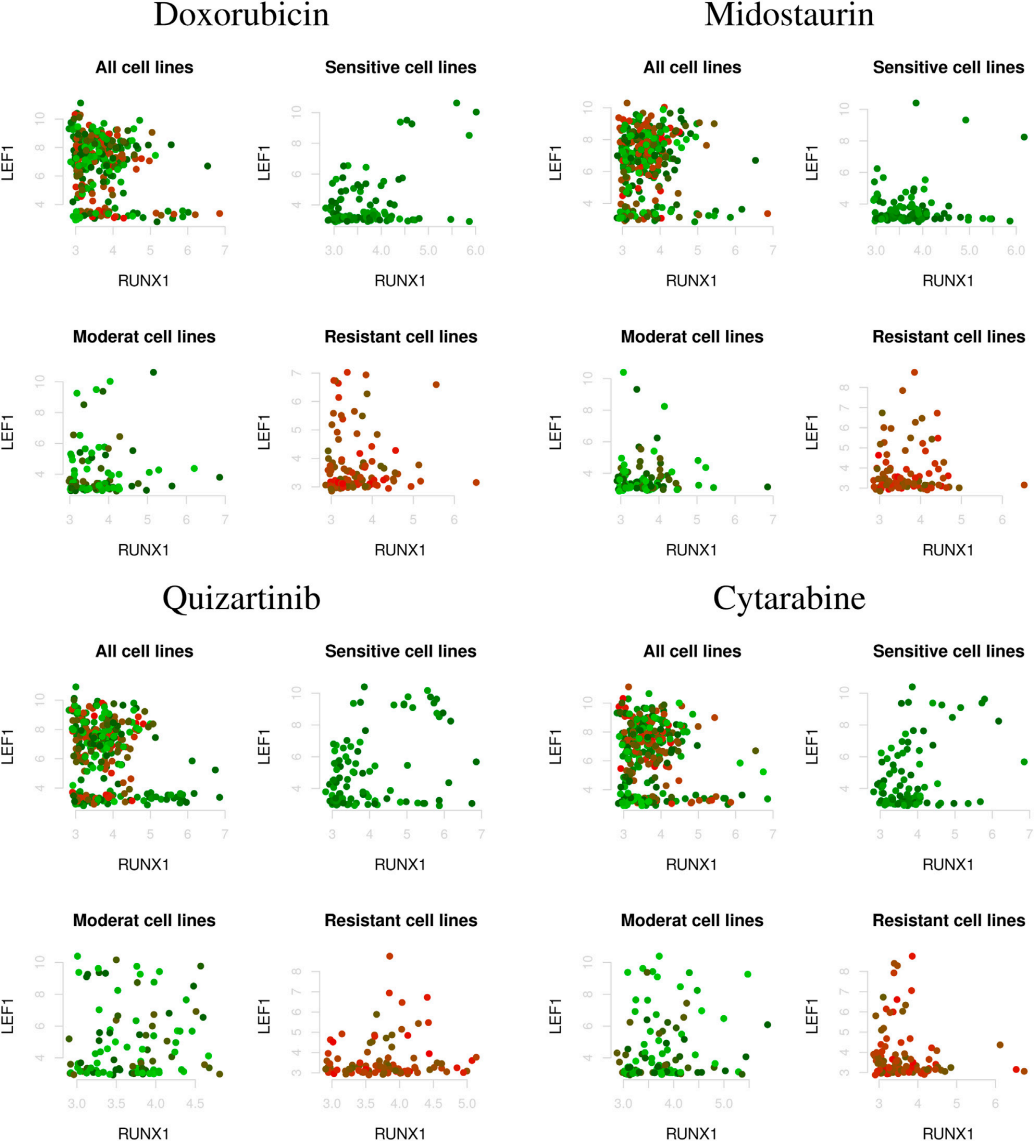
Correlations between two genes (i.e., LEF1 and RUNX1) under varying AML drug sensitivities; i.e., Z-score of IC50 values (top left: all cell lines; top right: drug-sensitive cell lines; bottom left: moderate-sensitive cell lines; bottom right: drug-resistant cell lines). The red and green dots indicate drug resistant and sensitive cell lines, respectively.

Figure 2 shows the correlations between two genes (i.e., LEF1 and RUNX1) that vary depending on AML drug sensitivity (i.e., as a characteristic of cell line), where the top left, top right, bottom left, and bottom right indicate the correlations between genes in all cell lines as well as drug-sensitive, moderate, and drug-resistant cell lines, respectively. As shown in Figure 2, the correlations between genes showed different patterns in the drug-sensitive and drug-resistant cell lines. However, the correlations in all cell lines did not capture drug sensitivity-specific patterns of association between the genes. This implies that gene regulatory networks should be estimated by considering the characteristics of the cell lines.

To address this issue and estimate a personalized gene network, we considered the following varying coefficient model (Hastie and Tibshirani, 1993),

$$y_{i\ell }=r_{i}^{T}\beta_{\ell }\left(m_{\alpha }\right)+\varepsilon_{i\ell },\;i=1,\ldots ,n,$$ (2)

where $\beta_{\ell }\left(m_{\alpha }\right)={\left(\beta_{1\ell }\left(m_{\alpha }\right),\ldots ,\beta_{p\ell }\left(m_{\alpha }\right)\right)}^{T}$ is the varying coefficient vector that describes the strength of the effects of p regulatory genes on the $\ell^{th}$ target gene in the network of the $\alpha^{th}$ target sample having a specific biological characteristic of cell lines, called a modulator $m_{\alpha }$ (e.g., drug sensitivity, cancer progression, etc.).

Shimamura et al. (2011) proposed the use of kernel-based $L_{1}$ -type regularization methods to estimate personalized gene networks (i.e., $\beta_{\ell }\left(m_{\alpha }\right)$ ),

$${\hat{\beta }}_{\ell \alpha }=\underset{\beta_{\ell \alpha }}{arg\;min}\left\{\frac{1}{2}\overset{n}{\underset{i=1}{\sum }}{\left(y_{i\ell }-r_{i}^{T}\beta_{\ell \alpha }\right)}^{2}K\left(m_{i}-m_{\alpha }|h_{\ell \alpha }\right)+P\left(\left|\beta_{\ell \alpha }\right|\right)\right\},$$ (3)

where $\beta_{\ell \alpha }=\beta_{\ell }\left(m_{\alpha }\right)$ and $P\left(\left|\beta_{\ell \alpha }\right|\right)$ denotes the elastic net penalty term (Zou and Hastie, 2005),

$$P\left.\left(\left|\beta_{\ell \alpha }\right|\right)\right\}=\lambda_{\ell \alpha }\overset{p}{\underset{j=1}{\sum }}\left[\frac{1}{2}\left(1-\pi_{\ell \alpha }\right)\beta_{\ell j\alpha }^{2}+\pi_{\ell \alpha }|\beta_{\ell j\alpha }|\right],$$ (4)

where $\lambda_{\ell \alpha }>0$ is a regularization parameter that controls the degree of shrinkage for $\beta_{\ell \alpha }$ , and $0\leq \pi_{\ell \alpha }\leq 1$ is a mixing parameter between the $L_{2}$ -norm [i.e., ridge (Hoerl and Kennard, 1970)] and $L_{1}$ -norm [i.e., lasso (Tibshirani, 1996)] penalties, and

$$K\left(m_{i}-m_{\alpha }|h_{\ell \alpha }\right)=exp\left\{\frac{-{\left(m_{i}-m_{\alpha }\right)}^{2}}{h_{\ell \alpha }}\right\},$$ (5)

is a Gaussian kernel function with the bandwidth $h_{\ell \alpha }$ . In kernel-based $L_{1}$ -type regularized regression modeling, the Gaussian kernel function plays a key role; that is, it measures the similarity between sample characteristics (i.e., ${\left(m_{i}-m_{\alpha }\right)}^{2}$ ), and then determines the amount of weight for each sample in gene network estimation of the $\alpha^{th}$ sample.

### 2.2 Generalized information criteria for personalized gene network analysis

In personalized gene network analysis based on kernel-based $L_{1}$ -type regularization, the selection of the regularization parameters (i.e., $\lambda_{\ell \alpha }$ and $\pi_{\ell \alpha }$ ) in Equation 4 is crucial because parameter selection can be considered edge selection and edge weight estimation. Furthermore, bandwidth $h_{\ell \alpha }$ selection is for Gaussian kernel function in Equation 5 vital in sample-specific analysis because the bandwidth controls the sample weighting. That is, too large a value of $h_{\ell \alpha }$ leads to ineffective sample-specific analysis results, whereas too small a value provides extremely small weights for almost all samples; both prevent proper gene network estimation.

In previous studies, cross-validation (CV) or traditional information criteria, e.g., AIC and BIC, have often been used to select the regularization parameters and bandwidth. However, CV leads to time-consuming results; in particular, personalized gene network analysis is based on n estimations of a model for each n sample; thus, it requires considerable computational complexity. In addition, traditional information criteria are not suitable for kernel-based $L_{1}$ -type regularized regression modeling because the criteria were derived under the assumption that the model is estimated using the maximum likelihood method (Konishi and Kitagawa, 1996; Konishi and Kitagawa, 2008).

In this study, we considered the generalized information criterion (GIC) for model evaluation of personalized gene network analysis (i.e., $\lambda_{\ell \alpha },\pi_{\ell \alpha }$ , and $b_{\ell }$ selection) (Konishi and Kitagawa, 1996). The GIC is derived by relaxing the following assumptions imposed on the AIC (Konishi and Kitagawa, 1996; Konishi and Kitagawa, 2008):

• The model is estimated by the maximum likelihood method.
• The estimation is carried out in a parametric family of distributions including the true model.

Thus, the GIC enables us to properly evaluate models estimated using various methodologies, not only the maximum likelihood method.

We derived a GIC for personalized gene network analysis based on a kernel-based $L_{1}$ -type regularization method. One of the key ideas for deriving GIC for the personalized gene network analysis is that the objective function of the kernel-based $L_{1}$ -type regularized regression model in Equation 3 can be represented without a Gaussian kernel function as follows:

$${\hat{\beta }}_{\ell \alpha }=\underset{\beta_{\ell \alpha }}{arg\;min}\left\{\frac{1}{2}\overset{n}{\underset{i=1}{\sum }}{\left(y_{i\ell }-r_{i}^{T}\beta_{\ell \alpha }\right)}^{2}K\left(m_{i}-m_{\alpha }|h_{\ell \alpha }\right)+P\left(\left|\beta_{\ell \alpha }\right|\right)\right\}=\underset{\beta_{\ell \alpha }}{arg\;min}\left\{\frac{1}{2}{\left(y_{\ell }-R\beta_{\ell \alpha }\right)}^{T}K_{\ell \alpha }^{T}K_{\ell \alpha }\left(y_{\ell }-R\beta_{\ell \alpha }\right)+P\left(\left|\beta_{\ell \alpha }\right|\right)\right\}=\underset{\beta_{\ell \alpha }}{arg\;min}\left\{=\frac{1}{2}{\left(y_{\ell }^{*}-R^{*}\beta_{\ell \alpha }\right)}^{T}\left(y_{\ell }^{*}-R^{*}\beta_{\ell \alpha }\right)+P\left(\left|\beta_{\ell \alpha }\right|\right)\right\}$$ (6)

where $R={\left(r_{1},\ldots ,r_{n}\right)}^{T}\in R^{n\times p}$ and

$$y_{\ell }^{*}=K_{\ell \alpha }y_{\ell }=\left[\begin{array}{ccc} k_{1\alpha } & & \\ & \ddots & \\ & & k_{n\alpha } \end{array}\right]\left[\begin{array}{c} y_{1\ell }\\ \vdots \\ y_{n\ell } \end{array}\right],$$

$$R^{*}=K_{\ell \alpha }R=\left[\begin{array}{ccc} k_{1\alpha } & & \\ & \ddots & \\ & & k_{n\alpha } \end{array}\right]\left[\begin{array}{ccc} r_{11} & \cdots & r_{1p}\\ \vdots & \ddots & \vdots \\ r_{n1} & \cdots & r_{np} \end{array}\right],$$

and where $k_{i\alpha }=\sqrt{K\left(m_{i}-m_{\alpha }|h_{\ell \alpha }\right)}$ . This Equation 6 implies that the personalized gene network is estimated using ordinary $L_{1}$ -type regularization methodology without the kernel function.

In the derivative of the GIC, the calculation of an influence function is crucial, where the second-order differentiable functional estimator ${\hat{\beta }}_{\ell \alpha }=T\left(\hat{G}\right)$ is required (Konishi and Kitagawa, 1996). For personalized gene network analysis based on the kernel-based $L_{1}$ -type regularization method, we estimate ${\hat{\beta }}_{\ell \alpha }=T\left(\hat{G}\right)$ as a solution to the system of implicit equations

$$\frac{\partial }{\partial \beta_{\ell \alpha }}\left\{\frac{1}{2}\overset{n}{\underset{i=1}{\sum }}{\left(y_{i\ell }^{*}-r_{i}^{*T}\beta_{\ell \alpha }\right)}^{2}+P\left(\left|\beta_{\ell \alpha }\right|\right)\right\}=0.$$ (7)

However, the estimator $\beta_{\ell \alpha }$ in Equation 7 cannot be derived analytically, owing to the indifferentiability of $L_{1}$ -type penalty as shown in Equation 4. To resolve this issue, we referred to the following local quadratic approximation (LQA) of an $L_{1}$ -type penalty (Fan and Li, 2001).

Suppose that we provide an initial value $\beta_{\ell \alpha 0}$ that is close to the minimizer of the objective function of the personalized gene network estimation in Equation 3. If $\beta_{j\ell \alpha 0}$ is close to 0, then ${\hat{\beta }}_{j\ell \alpha }=0$ . Otherwise, the $L_{1}$ -type penalty term can be approximated locally using a quadratic function as follows:

$${\left[P\left(\left|\beta_{j\ell \alpha }\right|\right)\right]}^{\prime }=P^{\prime }\left(\left|\beta_{j\ell \alpha }\right|\right)sgn\left(\beta_{j\ell \alpha }\right)\approx \left\{P^{\prime }\left(\left|\beta_{j\ell \alpha 0}\right|\right)/|\beta_{j\ell \alpha 0}|\right\}\beta_{j\ell \alpha },$$

when $\beta_{j\ell \alpha }\neq 0$ . Therefore,

$$P\left(\left|\beta_{j\ell \alpha }\right|\right)\approx P\left(\left|\beta_{j\ell \alpha 0}\right|\right)+\frac{1}{2}\left\{P^{\prime }\left(\left|\beta_{j\ell \alpha 0}\right|\right)/|\beta_{j\ell \alpha 0}|\right\}\left(\beta_{j\ell \alpha }^{2}-\beta_{j\ell \alpha 0}^{2}\right),$$

$\beta_{j\ell \alpha }\approx \beta_{j\ell \alpha 0}$ . Thus, Equation 7 can be approximated as follows:

$$-\overset{n}{\underset{i=1}{\sum }}\left\{y_{i\ell }^{*}-r_{i}^{*T}\beta_{\ell \alpha }\right\}r_{i}^{*}+\left\{P^{\prime }\left(\left|\beta_{j\ell \alpha 0}\right|\right)/|\beta_{j\ell \alpha 0}|\right\}\beta_{j\ell \alpha }=0.$$

This implies that the estimator ${\hat{\beta }}_{\ell \alpha }$ is given by ${\hat{\beta }}_{\ell \alpha }=T\left(\hat{G}\right)$ for the p -dimensional functional vector T(G) , which is defined as the solution of the implicit equation

$$\int \left[\left(y_{\ell }^{*}-r^{*T}T\left(G\right)\right)r^{*}+\left\{P^{\prime }\left(\left|T_{0}\left(G\right)\right|\right)/|T_{0}\left(G\right)|\right\}T\left(G\right)\right]dG=0.$$ (8)

To derive the following influence function $T_{\ell \alpha }^{\left(1\right)}$ , which is crucial to the derivative of GIC,

$$T^{\left(1\right)}\left(G\right)\equiv \frac{\partial }{\partial \varepsilon }T\left[\left(1-\varepsilon \right)G+\varepsilon \delta_{y}\right]{|}_{\varepsilon =0},$$

We substitute G with (1−ϵ)G+ϵδ in Equation 8, as follows:

$$\begin{array}{ll} & \int \left[\left(y_{\ell }^{*}-r^{T}T\left[\left(1-\epsilon \right)G+\epsilon \delta \right]\right)r^{*}+\left\{P^{\prime }\left(\left|T_{0}\left(G\right)\right|\right)/\times |T_{0}\left(G\right)|\right\}T\left[\left(1-\epsilon \right)G+\epsilon \delta \right]\right]\\ & d\left[\left(1-\epsilon \right)G+\epsilon \delta \right]=0. \end{array}$$ (9)

We then differentiate both sides of Equation 9 with respect to ϵ as follows:

$$\int [-r^{*}r^{*T}\frac{\partial }{\partial \varepsilon }T\left[\left(1-\varepsilon \right)G+\varepsilon \delta_{y}\right]-\left\{P^{\prime }\left(\left|T_{0}\left(G\right)\right|\right)/|T_{0}\left(G\right)|\right\}\frac{\partial }{\partial \varepsilon }T\left[\left(1-\epsilon \right)G+\epsilon \delta_{y}\right]]$$

$$d\left[\left(1-\varepsilon \right)G+\varepsilon \delta_{y}\right]$$

$$\;+\int [\left(y_{\ell }^{*}-r^{*T}T\left[\left(1-\varepsilon \right)G+\varepsilon \delta_{y}\right]\right)r^{*}-\left\{P^{\prime }\left(\left|T_{0}\left(G\right)\right|\right)/|T_{0}\left(G\right)|\right\}T\left[\left(1-\varepsilon \right)G+\varepsilon \delta_{y}\right]]$$

$$d\left(\delta_{y}-G\right)=0,$$

and set ε=0 . We then obtain the following Equation 10,

$$\int \left[-r^{*}r^{*T}-\left\{P^{\prime }\left(\left|T_{0}\left(G\right)\right|\right)/|T_{0}\left(G\right)|\right\}\right]dG\cdot \frac{\partial }{\partial \varepsilon }T\left[\left(1-\varepsilon \right)G+\varepsilon \delta_{y}\right]{|}_{\varepsilon =0}+\left(y_{\ell }^{*}-r^{*T}T\left(G\right)\right)r^{*}-\left\{P^{\prime }\left(\left|T_{0}\left(G\right)\right|\right)/|T_{0}\left(G\right)|\right\}T\left(G\right)=0.$$ (10)

Consequently, the influence function $T^{\left(1\right)}\left(G\right)$ of the functional that defines the kernel-based $L_{1}$ -type regularization estimator is given by the Equation 11,

$$\begin{array}{ll} T^{\left(1\right)}\left(G\right) & \equiv \frac{\partial }{\partial \varepsilon }T\left[\left(1-\varepsilon \right)G+\varepsilon \delta_{y}\right]{|}_{\varepsilon =0}\\ & ={\left[\int r^{*}r^{*T}+\left\{P^{\prime }\left(\left|T_{0}\left(G\right)\right|\right)/|T_{0}\left(G\right)|\right\}dG\right]}^{-1}\\ & \cdot \left[\left(y_{\ell }^{*}-r^{*T}T\left(G\right)\right)r^{*}-\left\{P^{\prime }\left(\left|T_{0}\left(G\right)\right|\right)/\left.\left|T_{0}\left(G\right)\right|\right\}T\left(G\right)\right\}\right]. \end{array}$$ (11)

Thus, the bias correction term in GIC for personalized gene network estimation is given as the following Equation 12,

$$b^{\left(1\right)}=tr\left({\left[\int r^{*}r^{*T}+\left\{P^{\prime }\left(\left|T_{0}\left(G\right)\right|\right)/|T_{0}\left(G\right)|\right\}dG\right]}^{-1}\right.\left.\int \left[\left(y_{\ell }^{*}-r^{*T}T\left(G\right)\right)r^{*}-\left\{P^{\prime }\left(\left|T_{0}\left(G\right)\right|\right)/|T_{0}\left(G\right)|\right\}T\left(G\right)\right]\cdot \frac{\partial logf\left(y_{\ell }^{*}|r^{*},\beta_{\ell \alpha }\right)}{\partial \beta_{\ell \alpha }^{T}}{|}_{\beta_{\ell \alpha }=T\left(G\right)}dG\right)+O\left(n^{-1}\right).$$ (12)

By replacing the unknown distribution G with the empirical distribution $\hat{G}$ and subtracting the asymptotic bias estimate from the log-likelihood, we can derive the GIC for the statistical model $f\left(y_{\ell }^{*}|r^{*},{\hat{\beta }}_{\ell \alpha }\right)$ with the functional estimator ${\hat{\beta }}_{\ell \alpha }=T\left(\hat{G}\right)$ as follows:

$$GIC=-2\overset{n}{\underset{i=1}{\sum }}logf\left(y_{i\ell }^{*}|r_{i}^{*},{\hat{\beta }}_{\ell \alpha }\right)+2tr\left\{R{\left(\hat{G}\right)}^{-1}Q\left(\hat{G}\right)\right\},$$ (13)

where

$$\begin{array}{ll} & R\left(\hat{G}\right)=\frac{1}{n}\left\{R^{*T}R^{*}+\Sigma_{\lambda }\left({\hat{\beta }}_{\ell \alpha }\right)\right\},\\ & Q\left(\hat{G}\right)=\frac{1}{n}\left\{R^{*T}{\hat{\Lambda }}^{2}R^{*}-\Sigma_{\lambda }\left({\hat{\beta }}_{\ell \alpha }\right){\hat{\beta }}_{\ell \alpha }1_{n}^{T}\hat{\Lambda }R^{*}\right\}, \end{array}$$

and where $\hat{\Lambda }$ and $\Sigma_{\lambda }\left({\hat{\beta }}_{\ell \alpha }\right)$ are n×n and p×p diagonal matrices, respectively,

$$\hat{\Lambda }=diag\left\{\left(y_{1\ell }^{*}-r_{1}^{*T}{\hat{\beta }}_{\ell \alpha }\right)/\sigma_{1}^{*2},\ldots ,\left(y_{1\ell }^{*}-r_{n}^{*T}{\hat{\beta }}_{\ell \alpha }\right)/\sigma_{n}^{*2}\right\},$$

$$\Sigma_{\lambda }\left({\hat{\beta }}_{\ell \alpha }\right)=diag\left[P^{\prime }\left(\left|\beta_{1\ell \alpha 0}\right|\right)/|\beta_{1\ell \alpha 0}|,\ldots ,P^{\prime }\left(\left|\beta_{p\ell \alpha 0}\right|\right)/|\beta_{p\ell \alpha 0}|\right],$$

and $\sigma_{i}^{*2}=k_{i\alpha }\sigma^{2}$ and $1_{n}={\left(1,1,,\ldots ,1\right)}^{T}$ are n -dimensional vectors. This implies that the GIC was derived without assuming maximum likelihood estimation. Thus, it can be applied to model evaluation for personalized gene network analysis based on various estimation methods.

Personalized gene network analysis is based on the selected tuning parameters $\lambda_{\ell ,\alpha },\pi_{\ell ,\alpha }$ , and $h_{\ell ,\alpha }$ , which minimize the derived GIC.

## 3 Monte Carlo simulation

Monte Carlo simulations were conducted to illustrate the performance of the proposed GIC in personalized gene network analysis.

Gene expression data were simulated under assumed personalized networks that varied depending on the characteristics of the samples. The expression levels of p -regulator genes were generated from a p -dimensional multivariate normal distribution, where the correlation between $r_{j}$ and $r_{k}$ was $\rho^{\left|j-k\right|}$ with ρ=0.5 . The expression levels of the $\ell^{th}$ target genes were calculated as the following Equation 14,

$$y_{i\ell }=r_{i}^{T}\beta_{\ell }\left(m_{\alpha }\right)+\varepsilon_{i\ell },\;i=1,\ldots ,n,$$ (14)

where $\varepsilon_{i\ell }∼N\left(0,1\right)$ and $M=\left(m_{1},\ldots ,m_{n}\right)$ are generated from a uniform distribution U(−1,1) .

We considered a sample size n=300 and a p -dimensional vector of coefficients consisting of a randomly selected 10% of variables with non-zero coefficients for 95% of samples (285 of 300 samples) and zero coefficients for 5% of samples. We then considered the remaining 90% of the regulator genes as noisy features (i.e., 90% of p variables have zero coefficients for all n samples). The nonzero-varying coefficients $\beta_{\ell \alpha }$ of the crucial 10% of variables were generated from various scenarios:

• Scenario 1:

$$\beta_{j\ell \alpha }=\left\{\begin{array}{cc} \text{are generated from}U\left(0.1,1\right),\; & \alpha =1,\ldots ,285,\\ 0,\; & otherwise. \end{array}\right.$$

• Scenario 2:

$$\beta_{j\ell \alpha }=\left\{\begin{array}{cc} \text{are generated from}U\left(0.9,1\right),\; & \alpha =1,\ldots ,285,\\ 0,\; & otherwise. \end{array}\right.$$

• Scenario 3:

$$\beta_{j\ell \alpha }=\left\{\begin{array}{cc} \text{are generated from}U\left(-1,-0.1\right),\; & \alpha =1,\ldots ,285,\\ 0,\; & otherwise. \end{array}\right.$$

• Scenario 4:

$$\beta_{j\ell \alpha }=\left\{\begin{array}{cc} \text{are generated from}U\left(-1,-0.9\right),\; & \alpha =1,\ldots ,285,\\ 0,\; & otherwise. \end{array}\right.$$

**FIGURE 3:**
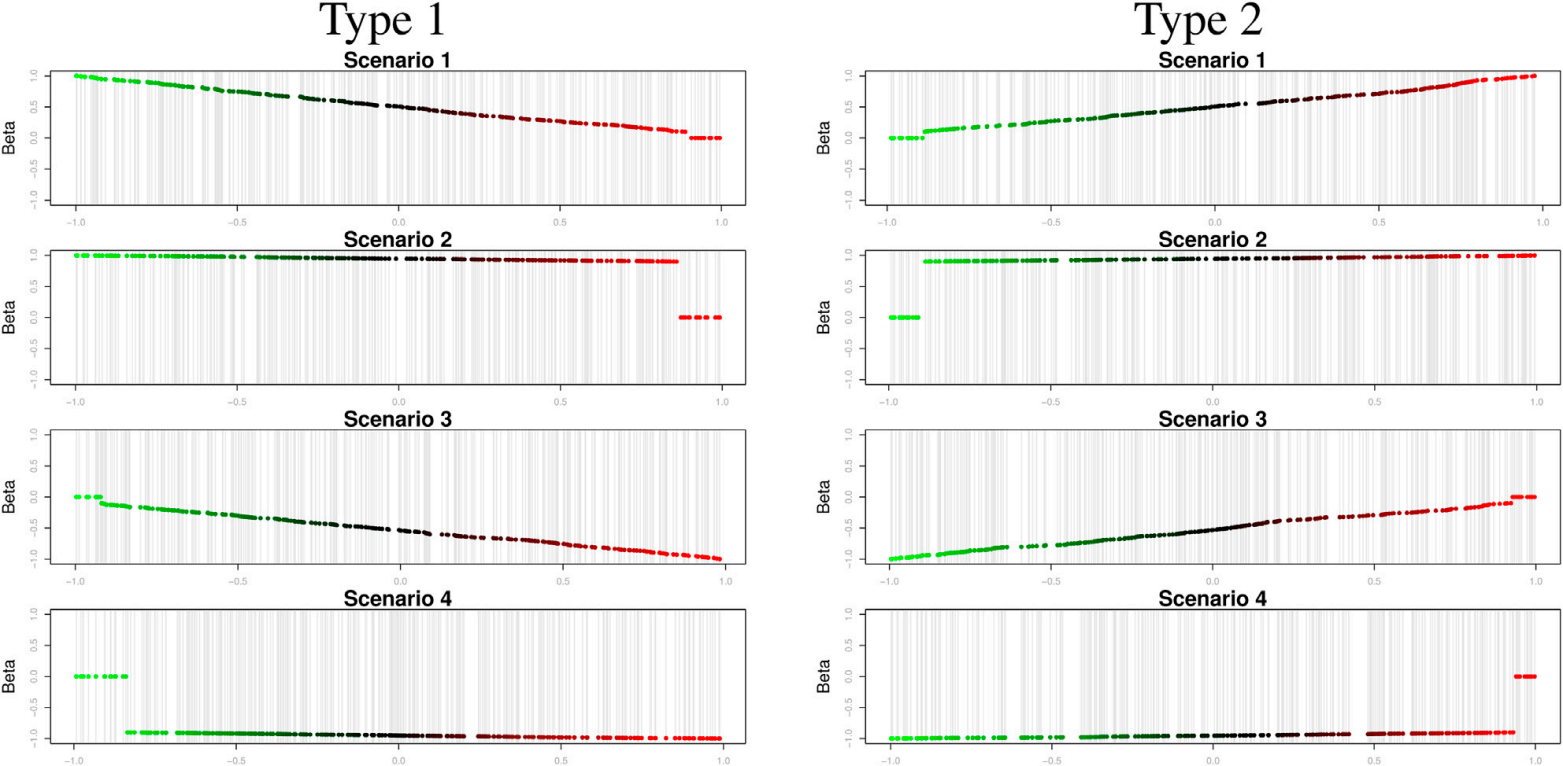
Varying coefficients to describe sample-specific edge weights.

Scenarios 1 and 2 (3 and 4) represent positive (negative) edge weights; that is, the strength of the effects of activators (inhibitors) on their target genes, where edge weights that vary greatly depending on the modulator values (i.e., $m_{i}$ ) are described in Scenarios 1 and 3. We also considered varying coefficients in descending and ascending order in simulation types 1 and 2. Figure 3 shows the varying coefficients to describe edge weights in personalized gene networks.

We considered the number of genes consisting of networks p+1 as 50, 100, and 500. Personalized gene networks were estimated for 40 randomly selected modulator values $M=\left(m_{1},\ldots ,m_{40}\right)$ .

The performance of the proposed model evaluation criterion (i.e., GIC) for personalized gene network analysis was evaluated by comparing it with CV and traditional information criteria, including AIC, BIC, Akaike’s second-order corrected Information Criterion (AICc) (Hurvich and Tsai, 1989), and the Hannah and Quinn Criterion (HQC) (Hannan and Quinn, 1979). CV was implemented using the R package *glmnet* (Friedman et al., 2024) and traditional information criteria were implemented using the R package*HDeconometrics* (Gabriel, 2016).We also show the evaluate results by the recently developed model evaluation criteria, i.e., extended BIC (EBIC) (Chen et al., 2022) and high-dimensional BIC (BIC-p) (Nan and Yang, 2014). The evaluation was conducted based on the accuracies of edge selection, including true positive (TP), true negative (TN), and their average values, based on 100 iterations. Table 1 lists the edge selection results, where bold numbers indicate the most effective performance among the model evaluation criteria.

**TABLE 1:** Accuracy of edge selection (true negative rate, true positive rate, and their average values), where bold numbers indicate the best performance among the model evaluation criteria, where “SN x ” indicate scenarios x .

Type		♯ genes: 50								♯ genes: 100								♯ genes: 500							
		SN1		SN2		SN3		SN4		SN1		SN2		SN3		SN4		SN1		SN2		SN3		SN4	
		1	2	1	2	1	2	1	2	1	2	1	2	1	2	1	2	1	2	1	2	1	2	1	2
True Negative Rate (TNR)	GIC	0.76	0.75	0.91	0.91	0.76	0.75	0.91	0.91	0.74	0.74	0.88	0.88	0.75	0.74	0.88	0.88	0.73	0.73	0.79	0.79	0.73	0.73	0.79	0.79
	BIC	0.81	0.80	0.77	0.79	0.81	0.80	0.77	0.78	0.73	0.73	0.67	0.67	0.73	0.74	0.68	0.67	0.72	0.73	0.76	0.76	0.72	0.72	0.76	0.76
	EBIC	0.87	0.88	0.90	0.90	0.88	0.87	0.91	0.90	0.87	0.88	0.88	0.88	0.87	0.87	0.88	0.88	0.97	0.96	0.88	0.87	0.96	0.96	0.88	0.88
	BICp	0.89	0.89	0.90	0.90	0.90	0.89	0.91	0.90	0.89	0.89	0.88	0.88	0.89	0.89	0.88	0.88	0.97	0.97	0.91	0.90	0.97	0.97	0.91	0.91
	AIC	0.27	0.28	0.27	0.27	0.28	0.28	0.28	0.29	0.16	0.17	0.15	0.15	0.16	0.17	0.16	0.15	0.68	0.68	0.74	0.74	0.68	0.68	0.74	0.74
	AICc	0.38	0.40	0.39	0.39	0.40	0.40	0.39	0.40	0.39	0.40	0.37	0.38	0.39	0.41	0.38	0.38	0.48	0.49	0.77	0.76	0.48	0.48	0.76	0.77
	HQC	0.57	0.59	0.56	0.57	0.58	0.58	0.56	0.56	0.46	0.47	0.43	0.44	0.46	0.47	0.44	0.43	0.69	0.69	0.74	0.74	0.68	0.68	0.75	0.74
	CV	0.73	0.72	0.71	0.71	0.73	0.73	0.71	0.72	0.73	0.73	0.73	0.71	0.73	0.73	0.71	0.72	0.72	0.73	0.70	0.70	0.71	0.71	0.70	0.70
True Positive Rate (TPR)	GIC	0.98	0.98	1.00	1.00	0.99	0.99	1.00	1.00	0.98	0.98	1.00	1.00	0.98	0.98	1.00	1.00	0.86	0.86	0.95	0.94	0.86	0.86	0.94	0.94
	BIC	0.94	0.95	1.00	1.00	0.94	0.94	1.00	1.00	0.96	0.95	1.00	1.00	0.96	0.96	1.00	1.00	0.84	0.85	0.96	0.96	0.84	0.85	0.96	0.96
	EBIC	0.88	0.88	1.00	1.00	0.87	0.89	1.00	1.00	0.81	0.81	1.00	1.00	0.83	0.81	1.00	1.00	0.16	0.17	0.59	0.60	0.19	0.17	0.59	0.58
	BICp	0.86	0.87	1.00	1.00	0.86	0.87	1.00	1.00	0.79	0.79	1.00	1.00	0.80	0.78	1.00	1.00	0.14	0.15	0.46	0.47	0.16	0.15	0.45	0.46
	AIC	0.99	0.99	1.00	1.00	0.99	0.99	1.00	1.00	1.00	0.99	1.00	1.00	0.99	1.00	1.00	1.00	0.87	0.87	0.96	0.96	0.88	0.88	0.96	0.96
	AICc	0.99	0.99	1.00	1.00	0.99	0.99	1.00	1.00	0.99	0.99	1.00	1.00	0.99	0.99	1.00	1.00	0.94	0.94	0.96	0.96	0.94	0.95	0.96	0.96
	HQC	0.98	0.98	1.00	1.00	0.98	0.98	1.00	1.00	0.99	0.99	1.00	1.00	0.99	0.99	1.00	1.00	0.87	0.87	0.96	0.96	0.87	0.88	0.96	0.96
	CV	0.96	0.96	1.00	1.00	0.97	0.96	1.00	1.00	0.96	0.96	1.00	1.00	0.95	0.96	1.00	1.00	0.84	0.84	0.97	0.98	0.85	0.85	0.98	0.98
Average of TPR and TNR	GIC	**0.87**	0.86	**0.95**	**0.96**	0.87	0.87	**0.96**	**0.96**	**0.86**	**0.86**	**0.94**	**0.94**	**0.86**	**0.86**	**0.94**	**0.94**	**0.80**	**0.79**	**0.87**	**0.87**	**0.80**	**0.80**	**0.86**	**0.87**
	BIC	**0.87**	**0.88**	0.89	0.89	0.87	0.87	0.89	0.89	0.84	0.84	0.84	0.84	0.84	0.85	0.84	0.84	0.78	**0.79**	0.86	0.86	0.78	0.79	0.86	0.86
	EBIC	**0.87**	**0.88**	**0.95**	0.95	**0.88**	**0.88**	0.95	0.95	0.84	0.84	**0.94**	**0.94**	0.85	0.84	**0.94**	**0.94**	0.56	0.57	0.73	0.74	0.58	0.57	0.73	0.73
	BICp	**0.87**	**0.88**	**0.95**	0.95	**0.88**	**0.88**	0.95	0.95	0.84	0.84	**0.94**	**0.94**	0.84	0.84	**0.94**	**0.94**	0.55	0.56	0.68	0.69	0.56	0.56	0.68	0.68
	AIC	0.63	0.64	0.64	0.64	0.64	0.64	0.64	0.64	0.58	0.58	0.58	0.58	0.58	0.58	0.58	0.58	0.77	0.78	0.85	0.85	0.78	0.78	0.85	0.85
	AICc	0.69	0.69	0.69	0.70	0.69	0.69	0.70	0.70	0.69	0.69	0.68	0.69	0.69	0.70	0.69	0.69	0.71	0.72	0.86	0.86	0.71	0.71	**0.86**	0.86
	HQC	0.78	0.78	0.78	0.79	0.78	0.78	0.78	0.78	0.72	0.73	0.71	0.72	0.72	0.73	0.72	0.72	0.78	0.78	0.85	0.85	0.78	0.78	0.85	0.85
	CV	0.84	0.84	0.86	0.86	0.85	0.85	0.86	0.86	0.85	0.85	0.86	0.85	0.84	0.84	0.86	0.86	0.78	0.78	0.84	0.84	0.78	0.78	0.84	0.84

As shown in Table 1, the proposed GIC and BIC-type criteria (BIC, EBIC, BIC-p) provide outstanding edge selection performance in personalized gene network analysis. Although EBIC and BIC-p show effective results, the methods cannot perform well for edge selection in high-dimensional situations (i.e., ♯ Genes: 500). The proposed GIC shows the most effective results compared with those of other traditional information criteria. Although other information criteria also show effective results for true edge selection, existing methods cannot perform well in terms of the true negative rate; in particular, AIC, AICc, and HQC show poor results. The performance of our strategy was also improved in scenarios with large absolute values of varying coefficients, as in scenarios 2 and 4, whereas the performance of other criteria did not improve.

**FIGURE 4:**
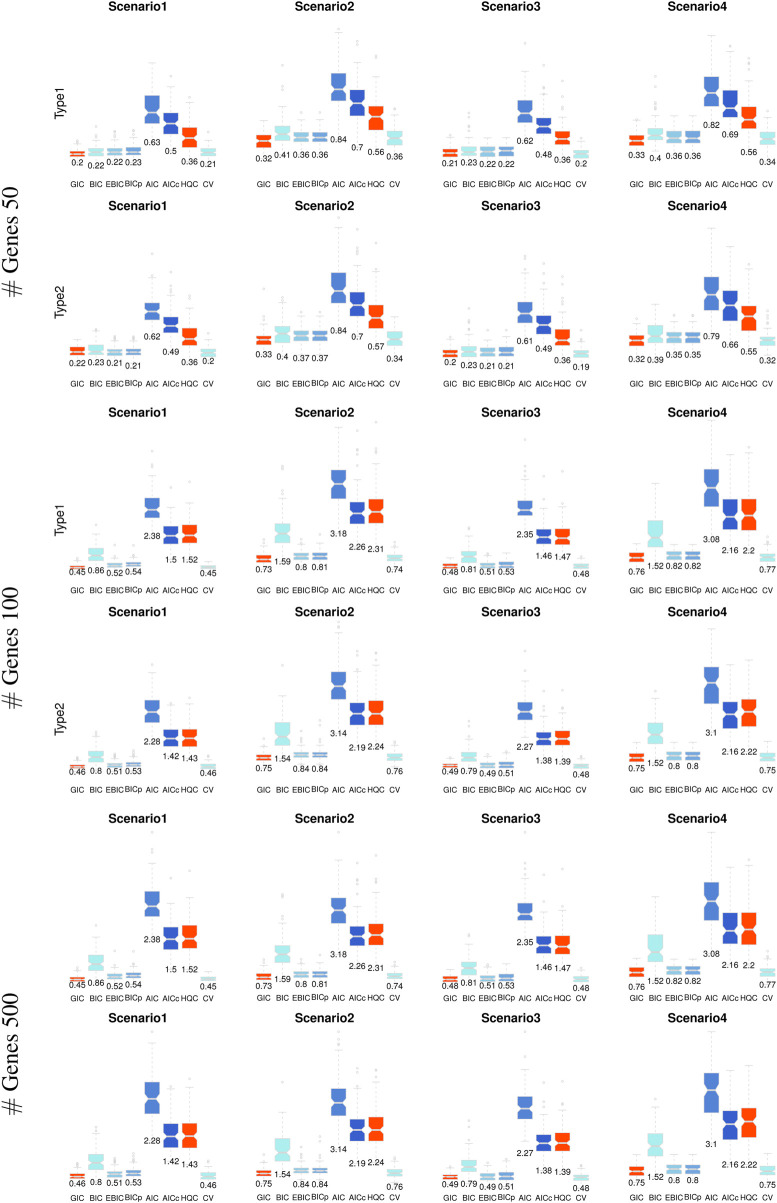
Mean absolute error of the edge weight estimation in personalized gene network analysis.

We also evaluated the accuracy of edge weight estimation based on the mean absolute error (MAE) of ${\hat{\beta }}_{\ell \alpha }$ as follows:

$$MAE\left({\hat{\beta }}_{\ell \alpha }\right)=\frac{1}{\omega }\overset{\omega }{\underset{\alpha =1}{\sum }}\overset{p}{\underset{j=1}{\sum }}|\beta_{j\ell \alpha }-{\hat{\beta }}_{j\ell \alpha }|,\;\ell =1,\ldots ,p,$$ (15)

where ω=40 denotes the number of target samples corresponding to the modulator values $M=\left(m_{1},\ldots ,m_{40}\right)$ . Figure 4 shows the MAE of the edge weight estimation in personalized gene network analysis in Equation 15. It can be seen through Figure 4 that the proposed GIC effectively performed edge weight estimation overall, although there were a few differences between the methods. CV also showed outstanding performance, particularly in gene network analysis with a large number of genes (i.e., # Genes 500) in scenarios 2 and 4. Our strategy also provided stable results, notably, with a small variance in the MAE, while AIC and AICc show especially poor results compared to those of other model selection criteria.

We also evaluated computational efficacy of the proposed GIC by compared with the CV. The varying coefficient model in Equation 2 for various data dimensional situations was considered, i.e., various number of regulator genes p=50,250,500,750,1000 and n=500 , where 40 target samples (i.e., $M=\left(m_{1},\ldots ,m_{40}\right)$ ) are considered. The computational efficiency is evaluated for modulator values in scenario for type 1, because computational cost is not affected by scenarios of modulator values. Table 2 shows computational times for the kernel-based $L_{1}$ -type regularized regression modeling with GIC and 10-fold CV.

**TABLE 2:** Computational costs in seconds for the kernel-based $L_{1}$ -type regularized regression modeling, where the hyper parameters are selected by GIC and CV. The computations are implemented by the glmnet R-packages.

Methods	♯ Genes: 50	♯ Genes: 250	♯ Genes: 500	♯ Genes: 750	♯ Genes: 1000
GIC	46.89	308.31	969.27	1195.34	2050.36
CV	109.27	1022.5	1430.52	1980.17	2309.81

As shown in Table 2, the proposed GIC provides computational cost-effective results compared with the CV. The challenge in computational complexity of GIC was computational of inverse matrix of $R\left(\hat{G}\right)$ in (13) and the problem becomes increasingly complex as the number of dimensions increases. Thus, the efficient computations of the inverse of a matrix should be considered for high-dimensional gene network analysis.

In summary, the proposed GIC effectively performed edge selection in personalized gene network analysis and provided efficient results for edge weight estimation. We expect that the proposed GIC will be a useful tool for model evaluation in personalized gene network analysis.

## 4 Anticancer drug sensitivity-specific gene network analysis

### 4.1 Acute myeloid leukemia drug sensitivity-specific gene network analysis

We applied the proposed GIC to AML drug sensitivity-specific gene network analysis. AML is a deadly hematopoietic malignancy characterized by the malignant proliferation of myeloid stem/progenitor cells (Culver-Cochran et al., 2024; Niu et al., 2022) Although the primary treatment for AML involves chemotherapy, acquired drug resistance in AML cell lines is a critical issue that leads to ineffective chemotherapy. Thus, uncovering the mechanisms underlying acquired AML drug resistance has been recognized as a critical problem. To uncover these mechanisms, we performed drug sensitivity-specific gene network analysis. We used the publicly available “Sanger Genomics of Drug Sensitivity in Cancer (GDSC) dataset from the Cancer Genome Project.” The gene expression and drug sensitivity data (i.e., the half-maximal inhibitory concentration (IC50) and its Z-score) were obtained from the GDSC dataset (https://www.cancerrxgene.org/). We considered four FDA-approved AML drugs, namely, doxorubicin, midostaurin, quizartinib, and cytarabine, which have sensitivity values in the GDSC dataset. We then considered 68 genes involved in the pathway “*Acute myeloid leukemia* (hsa05221)” of the KEGG pathway database (https://www.genome.jp/kegg/pathway.htm). For the 36 genes involved in the AML pathway that existed in the GDSC data, we extracted the expression levels of 300 randomly selected cell lines, including resistant (greater than $3^{rd}$ quantile of drug sensitivity), sensitive (smaller than $1^{st}$ quantile of drug sensitivity), and moderate (between the 40th and 60th percentiles of drug sensitivity) cell lines.

#### 4.1.1 Evaluation

**FIGURE 5:**
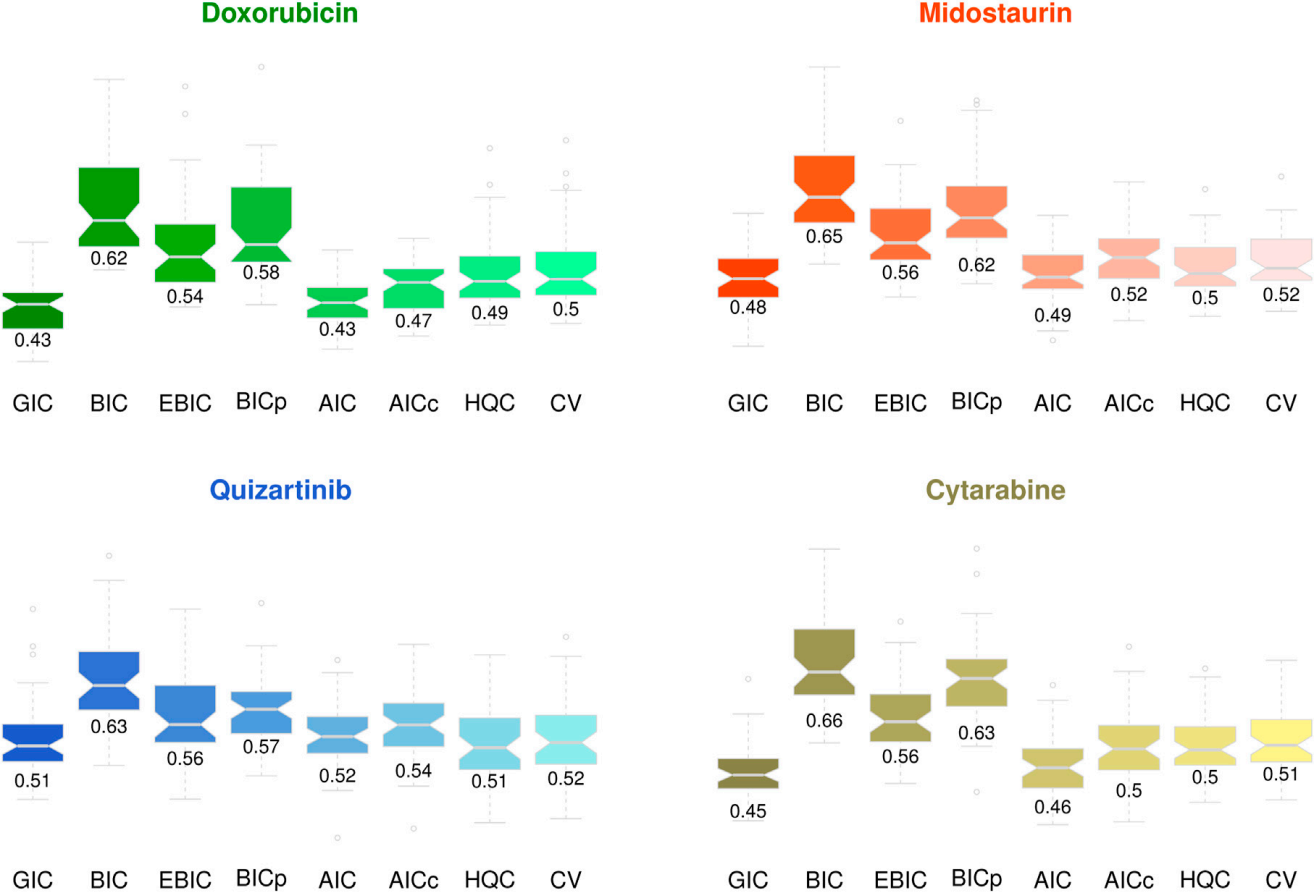
AML drug sensitivity-specific gene network estimation errors.

We first evaluated the performance of the proposed GIC based on an AML drug sensitivity-specific gene network analysis in which the Z-score of the IC50 value was used as a characteristic of the cell lines (i.e., modulator). Drug sensitivity-specific gene networks were estimated for a randomly selected set of each five sensitive, resistant, and moderately sensitive cell lines. We then evaluated the gene network estimation error, namely, the mean square error (MSE) of estimating the expression levels of the target genes based on the varying coefficient model (Equation 2). Figure 5 presents the average MSE over 50 iterations.

For doxorubicin, midostaurin, and cytarabine sensitivity-specific gene network analyses, the proposed GIC showed outstanding performance compared with that of other model evaluation criteria, whereas AIC also showed effective results in the quizartinib sensitivity-specific gene network estimation. Although there was no significant difference between the accuracies of the model selection criteria, the proposed GIC showed effective results in AML drug-specific gene network analysis.

#### 4.1.2 Uncovering AML drug resistant-specific molecular interactions

To uncover AML drug resistant-specific molecular interactions, we estimated drug sensitivity-specific gene networks for 100 randomly selected resistant and sensitive cell lines.

The medians of edge weights were computed for the 100 gene networks of 100 resistant cell lines. We then computed the means of the edge weights using four median edge weights from doxorubicin, midostaurin, quizartinib and cytarabine sensitivity-specific gene networks, where edges having non-zero median edge weights in the networks of the four drugs were only extracted. We defined the network based on the computed edge weights as the AML drug resistant-specific gene network. A similar process was conducted for the AML drug sensitivity-specific gene network.

**FIGURE 6:**
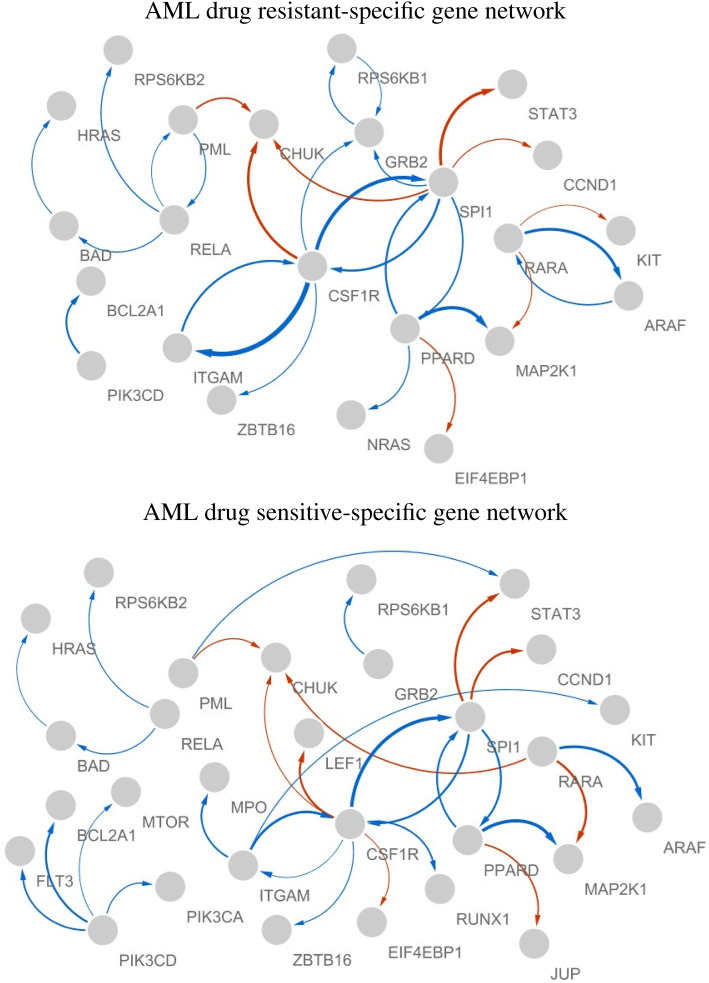
AML drug resistant- and sensitive-specific gene networks, where edge color indicates sign of the effect (red and blue are “-” and “+,” respectively), thickness represents the strength of the edge, and arrows (X → Y) indicate that gene X regulates gene Y.

Figure 6 shows the estimated AML drug-resistant- and sensitive-specific gene networks, where we considered only the largest 5% absolute edge weights for effective visualization.

In both AML drug resistant- and sensitive-specific gene networks, CSF1R, SPI1, and PPARD played key roles as hub genes. The activity of PIK3CD can be considered a drug sensitive-specific molecular interaction, whereas its activity becomes weaker in resistant cell lines. The hubness of RARA and RELA were AML drug resistant-specific molecular characteristic. Thus, CSF1R, SPI1, PPARD, PIK3CD, RARA, and RELA can be considered crucial biomarkers associated with the mechanisms of AML drug sensitivity. The markers identified in our analysis have been identified as crucial biomarkers of AML in literature, especially previous studies identified some markers as therapeutic targets of AML as follows.

 • CSF1R (Common marker)
       According to Edwards et al. (2019), inhibition of CSF1R, a receptor tyrosine kinase essential for the survival, proliferation, and differentiation of myeloid-lineage cells, demonstrated sensitivity. They identified CSF1R as a promising therapeutic target for AML and described its involvement in paracrine cytokine/growth factor signaling within this condition. CSF1R was suggested as an important target for sunitinib and related drugs (Kogan et al., 2012).
 • SPI1 (Common marker)
       Xiong et al. (2023) demonstrated that reduced circ-SPI1 expression correlates with lower white blood cell counts, favorable risk profiles, and enhanced therapy response, while its decrease during therapy independently predicts prolonged event-free and overall survival in patients with AML.
 • PPARD (Common marker)
       Lymboussaki et al. (2009) identified PPARD as a negative regulator of vitamin D3-induced monocyte differentiation, leading to the hypothesis that plays a role in the differentiation block observed in M5-type AML.
 • PIK3CD (Sensitive specific marker)
       Mutations in the AKT3 and PIK3CD genes were frequently observed in *de novo* Philadelphia chromosome-positive AML, highlighting the significant role of PIK3CD in cell proliferation and its potential as a therapeutic target for AML (Follo et al., 2019).
 • RARA (Resistant specific marker)
       de Botton et al. (2023) suggested that utilizing tamibarotene-based treatment in patients with AML or MDS and RARA overexpression might provide a personalized approach to achieving better therapeutic results. Fiore et al. (2020) suggested that SY-1425 plus azacitidine could serve as a novel targeted treatment option for RARA + newly diagnosed unfit AML, particularly for patients resistant to venetoclax-based standard-of-care therapy, warranting further exploration in this specific genomic subset. Stein et al. (2023) demonstrated that combining tamibarotene and azacitidine yielded a high response rate and rapid response onset with an associated favorable tolerability profile in newly diagnosed unfit patients with AML and RARA overexpression.
 • RELA (Resistant specific marker)
     RELA and PARP1 establish a positive feedback loop for DNA damage repair in AML cells, and inhibiting both NF- κ B and PARP1 boosts the antileukemic efficacy of daunorubicin *in vitro* and *in vivo*, highlighting the broader therapeutic potential of PARP1 inhibitors (Li et al., 2019). van Dijk et al. (2022) demonstrated that bortezomib could improve clinical outcomes in patients with AML and low levels of RELA-pSer536 and HSF1-pSer326.

**FIGURE 7:**
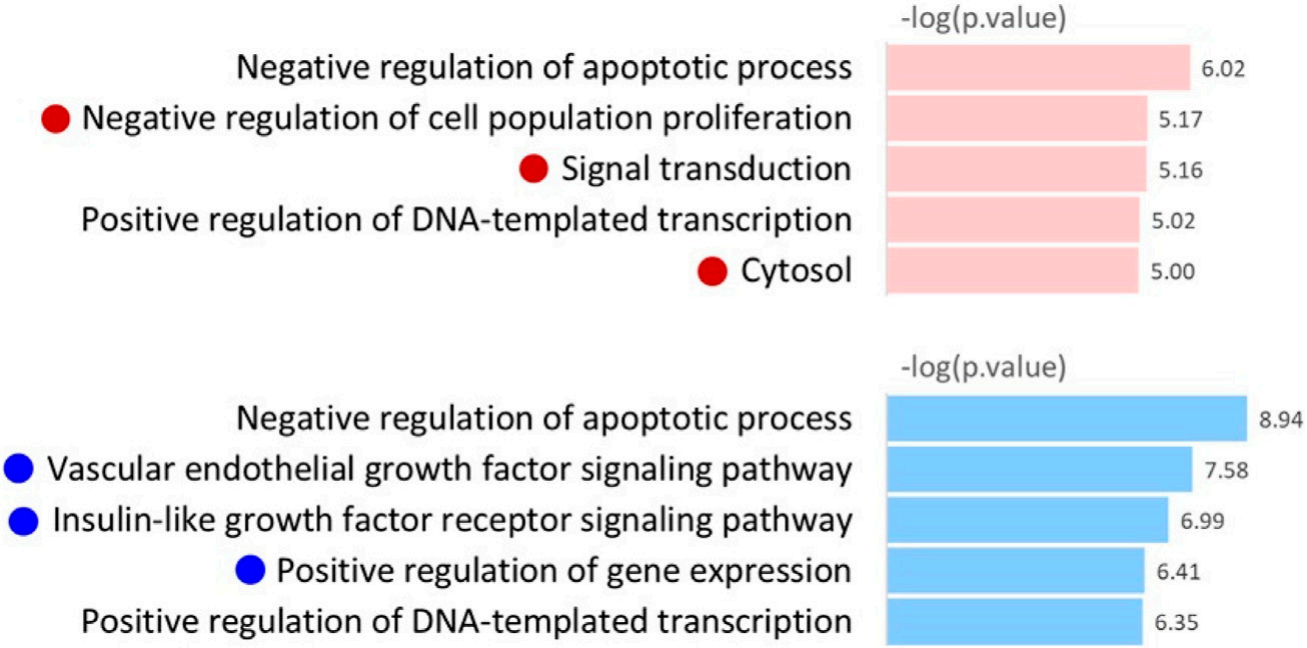
Gene Ontology analysis of AML drug resistant- and sensitive-specific gene networks.

To reveal the biological pathways and functions involved in AML drug resistant- and sensitive-specific gene networks, we performed gene enrichment analysis using the bioinformatics tool Database for Annotation, Visualization, and Integrated Discovery (DAVID) (Dennis et al., 2003). Gene Ontology (GO) analysis was performed using the categories “Molecular Function,” “Cellular Component,” and “Biological Processes.” The genes comprising the drug resistant- and sensitive-specific gene networks were used as inputs for GO term pathway analysis. Figure 7 shows the five most significant pathways with −log(p.value) .

As shown in Figure 7, the AML drug resistant and sensitive specific gene networks involve different biological pathways. The drug resistant-specific gene network was enriched in the *Cytosol Positive regulation of DNA-templated transcription, Signal transduction* and *Negative regulation of cell population proliferation* pathways. In contrast, *Positive regulation of gene expression, insulin-like growth factor receptor signaling pathway* and *Vascular endothelial growth factor signaling pathway* were identified as GO terms enriched in drug sensitive-specific gene networks. Furthermore, *Negative regulation of apoptotic process* and *Positive regulation of DNA-templated transcription* were identified as common GO terms enriched in both the drug resistant and sensitive specific gene networks.

Our results suggest that suppression of the identified AML drug resistant-specific markers (i.e., RARA and RELA) and activation of the sensitive-specific marker (i.e., PIK3CD) may be powerful means of improving chemotherapy efficacy in AML. Additionally, controlling the revealed AML drug resistant- and sensitive-specific pathways may help overcome drug resistance in AML.

### 4.2 Gastric cancer drugs sensitivity-specific gene network analysis

We also applied our strategy for gastric cancer drugs sensitivity-specific gene network analysis. We used the dataset obtained from the Cancer Dependency Map (DepMap) Portal (https://depmap.org/portal/), where the RNA expression levels were from the Cancer Cell Line Encyclopedia (CCLE) dataset and drug sensitivity measurements were obtained from the PRISM repurposing primary screen (https://depmap.org/repurposing). For the 148 genes involved in the gastric cancer pathway (i.e., *“Gastric cancer”* (hsa05226) of KEGG database) that existed in the CCLE data, we extracted the expression levels of 100 randomly selected cell lines. We focused on FDA approved gastric cancer drugs, 5-Fluorouracil, Capecitabine, Docetaxel, Doxorubicin, and Mitomycin-c. In the gastric cancer drugs sensitivity-specific gene network analysis, we consider a module of the drug sensitivities that describes common features of five drugs sensitivities. We then extracted the module of the gastric cancer drugs, i.e., we computed the first principal component of the drug sensitivities of 5 drugs. We then performed the gastric cancer drugs module-specific gene network analysis.

#### 4.2.1 Evaluation

**FIGURE 8:**
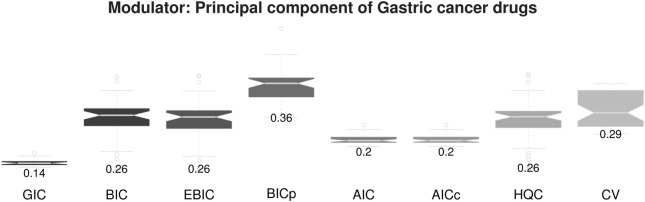
Gastric cancer drugs module sensitivity-specific gene network estimation errors.

We first evaluated our strategy based on MSE of the estimating expression levels of target genes, where randomly selected 10 samples having 5 largest and 5 smallest module values are considered as target samples. Our strategy was applied to hyper parameter selection in the kernel based $L_{1}$ -type regularized regression modeling. Figure 8 shows the average MSE over 50 iterations.

The proposed sample-specific GIC also provides outstanding performance for personalized gene network estimation. Furthermore, our strategy shows stables results compared with other methods, i.e., low variance of MSE. The result implies that the proposed method is a useful tool for personalized gene network analysis.

#### 4.2.2 Gastric cancer drugs sensitivity-specific molecular interplays

**FIGURE 9:**
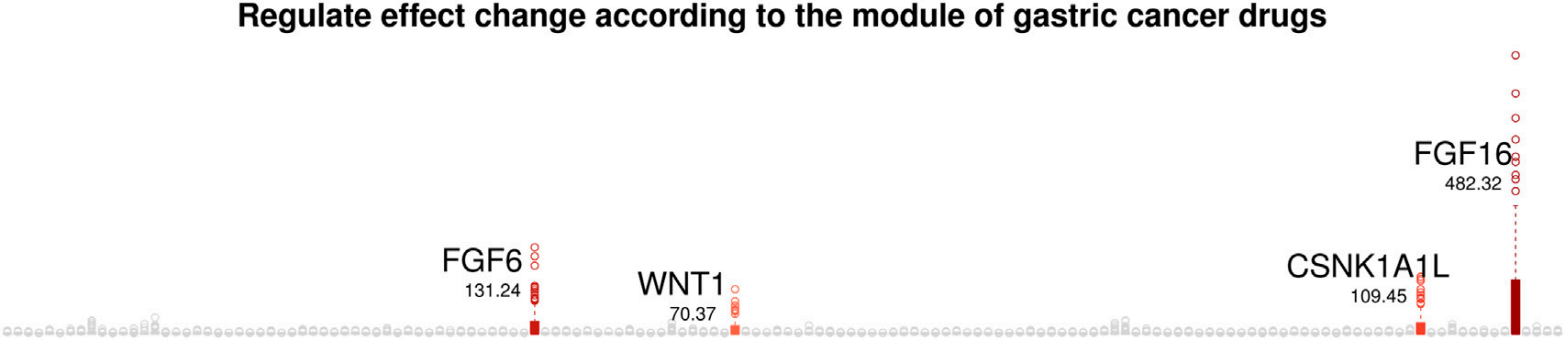
Regulate effect change of regulator genes according to the module of gastric cancer drugs.

We aim to uncover gastric cancer markers, i.e., candidate chemotherapy targets that have drug sensitivity specific molecular interplays. From the gastric cancer drug sensitivity module-specific gene networks, we computed effect change of regulator genes on their target gene according to ten modulator values, called a regulate effect. The regulate effect changes are computed as range of varying coefficients for 10 modules values. Figure 9 present the regulator effect changes of regulator genes on their target genes, where the numbers indicate total regulator effects for all target genes.

We focus on four genes, FGF16, FGF6, CSNK1A1L and WNT1 that show the largest regulate effect changes according to the module values of gastric cancer drug sensitivity. That is, FGF16, FGF6, CSNK1A1L and WNT1 show gastric cancer drug module-specific molecular interplays, and thus can be considered as candidate chemotherapy targets of gastric cancer.

 • FGF family (FGF16 and FGF6)
       Dysregulated FGF-FGFR signaling plays a major role in the onset of skeletal diseases and gastric cancer (Zhang et al., 2019). According to Zhang et al., 2021, FGF16 was found to be an immune-related gene with differential expression, significantly associated with overall survival in gastric cancer. Their study also highlighted the roles of NRP1, PPP3R1, IL17RA, and FGF16 in tumor progression and prognosis prediction.
 • CSNK1A1L
       CSNK1A1L, implicated in the Wnt signaling cascade, has been suggested as a diagnostic and prognostic marker in gastric and ovarian cancers (Anderson et al., 2015; Yang et al., 2017; Seabra et al., 2014 further demonstrated that CSNK1A1L expression varies across tumor stages, with notable differences between T4 and T1–T3 stages.
 • Wnt1
       Dou et al. (2020) demonstrated that dysregulation of the cell cycle by Wnt1 plays a critical role in driving ovarian cancer development. The study by Wang and Gao (2021) revealed that H19 promotes ovarian cancer progression by sequestering miR-140, which in turn leads to Wnt1 upregulation and increased cell proliferation and migration. Li et al., 2023 found that WNT1 expression is significantly upregulated in gastric cancer tumors. Their findings also indicate that KLF3 may enhance tumor progression and metastasis by stimulating the WNT/ β -catenin signaling cascade via WNT1. Mao et al., 2014 demonstrated that elevated Wnt1 and CD44 expression correlates with higher gastric cancer grades.

**FIGURE 10:**
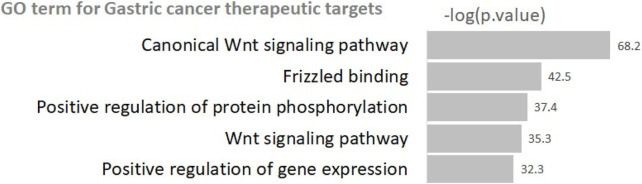
Gene Ontology terms for gastric cancer therapeutic targets.

Figure 10 shows the most significant GO terms for the identified gastric therapeutic targets (i.e., FGF16, FGF6, CSNK1A1L and WNT1) and their target genes.

The results indicate that the identified therapeutic targets are involved in Wnt signaling-related pathways (i.e., *Wnt signaling pathway* and *Canonical W. signaling pathway*). Abnormal regulation of Wnt pathway components has been observed in gastric cancer cells, contributing to uncontrolled cell growth, increased invasiveness and metastasis, poor clinical outcomes, and resistance to chemotherapy (Han et al., 2024). Furthermore, positive regulation-related terms (i.e., *Positive regulation of gene expression* and *Positive regulation of protein phosphorylation*) are also identified as GO terms enriched in the identified markers. It can be suggested through our results and literature survey that the identified genes (FGF16, FGF6, CSNK1A1L, WNT1) and Wnt signaling-related pathways provide crucial clue to chemotherapy efficacy of gastric cancer.

## 5 Discussion

In this study, we introduce a novel model evaluation tool for personalized gene network analysis. Although the kernel-based $L_{1}$ -type regularization methodology has been used to estimate sample-specific gene networks, relatively little attention has been paid to model evaluation of sample-specific analysis (i.e., regularization parameters and bandwidth selection). Previous studies have used CV or traditional information criteria (e.g., AIC and BIC, etc.) to evaluate personalized models. However, CV suffers from computational complexity and is thus unsuitable for personalized gene network analysis based on n estimations of models. Furthermore, traditional information criteria were derived under the assumption that the model is estimated using the maximum likelihood method. Thus, traditional information criteria cannot properly perform personalized gene network analysis using a kernel-based $L_{1}$ -type regularization method.

To address these issues, we proposed a GIC for personalized gene network analyses. Because the GIC was derived by relaxing the assumptions that *1. The model was estimated using the maximum likelihood method* and *2. The estimation was carried out in a parametric family of distributions, including the true model*, it properly evaluated the models for personalized gene network analysis. To derive the GIC, we first focused on the objective function of the kernel-based $L_{1}$ -type regularization method, which can be represented without a kernel function. Subsequently, to address the indifferentiability of the $L_{1}$ -type penalty in the computation of the influence function of the GIC, we referred to the local quadratic approximation of the $L_{1}$ -type penalty term and derived the GIC for personalized gene network analysis.

Monte Carlo simulations were conducted to demonstrate the performance of the proposed model evaluation strategy. Experiments with synthetic data demonstrated that the proposed GIC provided superior performance for edge selection in personalized gene network analysis. Furthermore, our strategy demonstrated effective results for edge weight estimation. We applied the proposed GIC to AML drug sensitivity-specific gene network analysis for FDA-approved AML drugs, including doxorubicin, midostaurin, quizartinib, and cytarabine. Our strategy yielded efficient network estimation results. From AML drug resistant- and sensitive-specific gene network analysis, we revealed that PIK3CD and RARA/RELA are sensitive- and resistant-specific markers, respectively. We suggest that RARA and RELA suppression and PIK3CD activation may provide crucial targets for improving chemotherapy efficacy in AML. We expect that the proposed strategy will be a useful tool not only for personalized gene network analysis, but also for various sample characteristic-specific analyses.

Although our strategy showed effective results for personalized gene network analysis, there are several limitations.

 • Asymptotic bias of $L_{1}$ norm penalty
       To calculation of the influence function in GIC, we use the LQA of $L_{1}$ norm penalty. Unfortunately, the LQA suffers bias because the technique is based on the Taylor series expansion. That is, there is a bias between the true function and the quadratic approximation of the $L_{1}$ norm penalty. Although the LQA is used for derivative of GIC to model evaluation and thus not lead to biased edge weight estimation in our strategy, evaluation of the estimated gene network suffers from the asymptotic bias. The employing bias-corrected approximation in derive GIC is considered as one of future work of our strategy.
 • Applicability for categorical sample characteristic (e.g., tumor subtypes) analysis
       The proposed strategy cannot be applied to categorical sample characteristic (e.g., tumor subtypes) analysis, because the kernel-based $L_{1}$ -type regularization is based on Gaussian kernel function. We consider extension of our strategy to categorical sample characteristic-specific gene network analysis based on kernel functions of categorical variables (Belanche and Villegas, 2013) as another future work of current study.
 • Lack of experimental validation
       In this study, we identified CSF1R, SPI1, PPARD, PIK3CD, RARA, and RELA as crucial AML markers by data-driven strategy and the identified markers were validated through literature survey. However, the literature survey is not enough to support biological evidences of our results. Although our study focuses on a computational strategy for personalized gene network analysis, the lack of experimental validation can be considered as one of limitation of this study.

Although we performed personalized gene network analysis focused on the anti-cancer drug sensitivity of samples, our strategy can be extended to various sample characteristics-specific analysis with continuous sample characteristics (e.g., drug sensitivity, cancer progression, survival time). Especially in the medical field, survival analysis plays a pivotal role in examining how outcomes evolve over a period. We consider application of our strategy for survival time specific gene network analysis and uncovering crucial molecular interplays influencing survival time dynamics as one of future work of this study.

## Data Availability

The original contributions presented in the study are included in the article/supplementary material, further inquiries can be directed to the corresponding author.
